# Levers of change: using mathematical models to compare gender equity interventions in universities

**DOI:** 10.1098/rsos.220785

**Published:** 2022-09-07

**Authors:** Alex James, Ann Brower

**Affiliations:** ^1^ School of Mathematics and Statistics, University of Canterbury, Aotearoa, New Zealand; ^2^ School of Earth and Environment, University of Canterbury, Aotearoa, New Zealand

**Keywords:** gender equity, Leslie matrix, management practices

## Abstract

Women are under-represented in academic staff in universities worldwide. Our work builds on other studies of ‘demographic inertia'. We find that time will not bridge the gender representation gap in academia, and echo others in saying bold actions are required to reach parity. Our work then uses New Zealand's unique system of scoring individual research performance to test empirically which levers universities should pull, and in which combinations. We combine individual research performance scores with 20 years of data from one university to parametrize a rank-structured mathematical model using Leslie matrices. Our model compares three key levers of change at universities' disposal—hiring, promotion and attrition. We apply the model to a bifurcated population of university staff—those with high research activity, and those who are moderately active—based on their national research quality score. We then test levers in various combinations that management could pull to improve gender representation. We find that the solutions are different for the high versus moderate research performers. For individuals with high research activity, universities should concentrate on equitable hiring practices. For those with more moderate research activity, more equitable promotion practices hold the key.

## 1. Introduction

The gender representation gap in academia, i.e. the lack of women in comparison with men, is widely lamented [1,2]. The gap still exists despite women comprising more than 50% of postgraduate students in many disciplines for many years [3,4]. The women who do work in the academy are clustered at the lower ranks of e.g. lecturer (L) and senior lecturer (SL) in the British system, rather than the upper levels of associate professor (AP) and full professor (P) [5]. This over-representation of women in the lower ranks, and therefore pay grades, is one of the contributors to the gender pay gap in universities.

Some studies discuss solutions for the gender pay gap, but there are few large-scale quantitative studies [6]. In this article, we recognize that without also fixing the gender representation gap, we will never achieve equality. For example, an institution with only 20% women might have pay equity if those women and men are distributed across ranks in the same pattern; but women will still be under-represented at that university even if they are well paid. That said, if we were to narrow the gender representation gap at every rank, we would also succeed in narrowing, and possibly eliminating, the gender pay gap.

Addressing the representation gap has benefits beyond pay equity. It has long been recognized in research and development that diversity leads to more innovation [7] and non-sexist academic environments lead to positive job outcomes [8]. A higher number of women academics leads to a wider range of role models for students [2] giving rise to a positive feedback that encourages diversity.

Demographic inertia, where a time lag is expected before change is seen [9] was once often cited as a reason for the lower representation of women particularly at higher levels [10]. But waiting for the passage of time was debunked as a strategy to achieve gender parity in representation [11,12]. Although inertia is now recognized as being only part of the problem [13], it is still clearly playing a part [9]. Previous studies of demographic inertia argued that gender parity in academia is ‘not inevitable' [14], even with the passage of time [15], and argued that bold interventions are required [11,14,15]. Our study agrees with, and builds on, these studies by empirically testing the levers that those interventions might pull to achieve gender parity in representation. Indeed, at our own institution, the University of Canterbury, Aotearoa, New Zealand, the proportion of staff who are women, particularly at P rank, has increased from fewer than 3% in 2005 to 25% in 2020 but is still far from parity.

Yet the following questions remain: (i) whether, if we continue with our current patterns and trends, representation equity will continue to improve until women reach parity; (ii) whether and when parity will be reached at all ranks and cohorts; (iii) whether and when parity will be reached for all high and moderate performers; (iv) which levers at universities' disposal would be most effective to ‘pull' to enhance equity. To explore these questions, we create a mathematical model of the number and rank distribution of academic staff at a university and parametrize it by combining two datasets: (i) a national dataset that scores each individual academic staff member's research output and impact on a scale from 0 to 700 points, with (ii) 20 years of anonymised HR data from one university.

The model uses Leslie matrices and compares three key drivers, or levers, that universities could pull or tweak: hiring, promotion and attrition. A difference in any one of these drivers will lead to a difference in the final make-up of staff. However, the interplay of these effects can make a different set of inputs lead to the same outcome. For example, if women were predominantly hired at lower levels than men, it is possible this could be offset by improved promotional prospects and lower attrition. In other words, gender differences in inputs could still produce equal representation. We focus our analysis on these three levers to provide a thorough understanding so that management decisions and programmes (such as women mentoring schemes with a focus on improving promotion rates and menopause awareness training to slow attrition) can be targeted to where they will be most helpful.

Exploring these three levers of change is difficult to do. It is hard for studies to qualify the effects of these interventions as, like any social science phenomenon, individual behaviour is hard to quantify. Instead, our approach is one of applying the well-established mathematical modelling tools of Leslie matrices [16], primarily developed for ecological populations, to the population dynamics of academics. Similar approaches have been used in the past. For example, Shaw & Stanton [9] examine the entire academic pipeline from undergraduate to P to establish bottlenecks that limit women's progression. Danell & Hjerm [17] use a promotion model to conclude that women's promotion rates from PhD completion to P are less than men's. Clifton *et al*. [14] use a dynamical model to assess levels of bias and homophily in different academic fields, also concluding that parity will only be reached with intervention in many areas. Finally, Lawrence & Chen [18] compared two departments within a single university focusing on the overall proportion of women rather than considering each rank separately as we do. Furthermore, our model is the first approach of this type to split the population by research activity level, recognizing the different career tracks that can be taken by individuals in the same institution.

To summarize, we use a simple Leslie matrix model, structured by academic rank, to predict the future size and gender representation patterns of the academic population. Our model uses three key inputs which are all easily extracted from payroll data: (i) the expected number of new hires at each academic rank, (ii) the probability of being promoted to the next rank in the annual promotion process, and (iii) the probability of an individual leaving the institution given their rank. By extracting these parameters separately from the data for men and women academics, we get two independent models whose outputs can be combined to predict the long-term gender representation at each rank. We run the model on three separate cohorts or populations: all academics, academics with moderate research activity and academics with high research activity. For each cohort, we parametrize the model from 20 years of data, validate the model by comparing the last 15 years of data with our model prediction and predict the long-term gender representation, i.e. the model steady state. Finally, we use the model to explore a range of management scenarios. As the three key inputs are independent in the model formulation, we can manipulate each one both separately and in combination with the others to see the effect of improving equity in any particular input. For example, if men's and women's promotion rates were identical, does this significantly improve the gender representation gap? Is attrition a more important driver? Or is simply hiring more women the key to fixing the gap?

## 2. Data

We used two datasets: payroll data supplied by the University of Canterbury, Aotearoa, New Zealand and Performance Based Research Fund (PBRF) scores supplied by the Tertiary Education Council of New Zealand (TEC). The bulk of the analysis was done using the detailed data from University of Canterbury (UC), and the TEC dataset was used to provide some national context to this particular university.

### 2.1. University

We use 12 855 records, anonymized, containing information for over 1300 academics (including 478 women) employed on a continuing academic contract, equivalent to tenured in the US system, at the UC between 2000 and 2020 for every pay step at which they were employed. The dataset does not include graduate students, postdoctoral research fellows or teaching only Ls. Each record includes start date, end date, employee ID number (anonymized) and step on the pay scale. From this data, it is possible to retrieve the pay step an individual was hired at (provided they were hired after 2000), the pay step they left at (provided they left before 2020) and any rank promotions during that time.

Like most New Zealand (NZ) universities, there are four major academic ranks: L, SL, AP and P. Individuals must apply to be promoted from one rank to the next. In general, all individuals move up a single pay step each year until the highest step in that rank is achieved. Tertiary Education Union [19] gives details on individual steps and ranks. Each rank contains a defined number of pay steps. Upon a successful promotion application, they then move to the next rank. It is no longer uncommon for individuals to be promoted before they reach the top step in that rank. It is also not uncommon for individuals to sit at the top step within a rank for many years. For promotion from L to SL, an applicant must excel in any two of the primary areas of teaching, research and service; but at the higher ranks, strong research performance is almost always required.

Additional information for every individual containing gender, date of birth, ethnicity and academic department or school for all individuals was also supplied.

### 2.2. National data

We combine the UC data with a dataset unique to Aotearoa New Zealand—the PBRF. This national exercise gives every New Zealand academic a personal research score between 0 and 700 based on a holistic assessment of their research outputs from the previous 6 years. This allows us to group staff into groups of high research activity and moderate research activity, to consider if different levers are required for academics on different career pathways.

National research performance assessments were carried out in 2006, 2012 and 2018. These are similar to the UK Research Excellence Framework but scores are awarded to individuals rather than groups. Any academic employed at the census date was given a Research Performance Score based on a holistic assessment of their research outputs in the preceding 6 years. These assessments are done by external panels of local and international experts with specific knowledge of that particular research area. Scores are cross-referenced between panels to ensure consistency between academic disciplines. Scores range from 0 to 700. The data included score, anonymised ID number, institution, date of birth, ethnicity, gender and research field.

To assign each individual employed by UC with their highest research score during their employment without using names, we matched the TEC dataset with the UC data using institution, date of birth, gender and ethnicity. When further clarification was needed, we also used the research area of the PBRF assessment panel and UC academic department.

As with any performance scoring mechanism, the PBRF is neither free of bias nor immune from ‘gaming the system' [20]. Women's average scores have been consistently 5–10% lower than men's since the scheme's inception [6], and one study observed women disproportionately experience effects of PBRF in their lived experience of academic life [21]. Further, researchers of both European and indigenous Maori descent have argued that the scheme undermines scholarship by and about indigenous Maori [22,23]. Recent changes to the PBRF scoring processes for the next round (in 2024) aim to address some of these biases (both conscious and unconscious) that have led to what Locke and Bensky called a ‘regime of inequality' [24]. This article does not address the debates surrounding PBRF, instead uses the dataset to explore questions of employment equity.

## 3. Grouping individuals by research score

For our analysis, we use each individual's highest recorded PBRF score across all three assessments (2003/6, 2012, 2018) as a proxy for their long-term research activity. It should be noted that PBRF scores are grouped into ‘grades' as follows: 600–700 is A grade (about 15% of the population); 400–599 is B grade; 200–399 is C grade; and 1–199 is R or ‘research inactive'. Among UC staff, very few had a highest score less than 200; and indeed nationally, the number of R grades among university staff has decreased dramatically since the inception of PBRF in 2003 [25].

As such, we use the mid-point of the B grade scores as the cut-off between high and moderate research performance; this is a score of 500. This puts the highest third of researchers into the high activity group. We classified individuals with a maximum research performance score of less than 500 as having moderate research activity (354 individuals, including 136 women). Individuals with a score equal to or higher than 500 were classified as having high research activity (220 individuals including 59 women). The remaining individuals did not have a research score, but were included in analysis on all individuals. The majority of individuals without research scores were hired after 2018 (the latest PBRF assessment round), though some left before 2003 (the first PBRF assessment round) or were only employed for a short period that did not coincide with an assessment census date (e.g. 2013–2017).

We use highest score as a proxy for an individual's research and hence career potential. Scores usually increase fairly rapidly at the start of an individual's career then plateau later on. Using an individual's highest research score has the possibility of misclassifying individuals, particularly if they only have a single score available, for two different reasons: (i) national level changes in scores between assessment rounds and (ii) personal changes in score as individuals progress in their careers.

### 3.1. Score changes between assessment rounds

Mean research performance scores, both nationally and at UC, showed a large increase between 2006 and 2012 (table 1). Nationally the expected score jumped from 338 points (out of a possible 700) to 418, a rise of over 23%. This increase stabilized between 2012 and 2018 when the expected score was 433, a rise of less than 4%. This larger initial increase probably reflects the bedding in of a new system as individuals, universities and the governing body TEC familiarized themselves with the scoring system. This stabilization is also seen, though not as dramatically, in the UC scores (table 1). The largest score increases are seen by researchers at the lower ranks of L. Researchers at the higher ranks of P and AP saw very small score increases, particularly between 2012 and 2018. Again, UC follows this national pattern.

**Table 1.** Mean research performance scores by rank.

	mean score of NZ researchers by rank			mean score of UC researchers by rank		
	2006	2012	2018	2006	2012	2018
L	223	291	309	248	294	316
SL	327	371	387	382	388	392
AP	483	485	490	502	481	468
P	511	563	573	507	554	565
all	338	418	433	376	416	438

As research scores were much lower in the 2006 PBRF assessment than in later rounds, using only an individual's highest score risks underestimating the activity of individuals who only entered this first assessment round. For all other individuals, who entered either 2012 and 2018 rounds or both, using just one score is expected to give a close approximation of how their research activity is perceived by others throughout their career.

### 3.2. Score changes due to career progression

Previous analysis on individual scores [6] showed that an individual's score is expected to rise at the start of their career, peak at around age 50 then decrease slightly. This means that early career individuals, particularly those with only a single score may be misclassified as having moderate research activity when actually they have high activity. We test this misclassification in the sensitivity analysis by using a different score cut-off for younger individuals. In the sensitivity analysis, we also test a classification scheme using an individual's average score, instead of their maximum score, across all assessment rounds.

## 4. A national context for the study of a single institution

We use the University of Canterbury, Aotearoa, New Zealand, to parametrize and validate the model. Studies on a single institution are often easily invalidated, as they are just a single data point. We offset this criticism by using national data on rank and research activity to set this one institution into a national context. As such we show that UC is not an outlier in any respect, and is probably representative of at least this country. In international terms, NZ is also rarely seen as an outlier. Despite its claim to be the first to give women the vote, it is not the haven of gender equity that some Scandinavian countries can claim to be, nor does it stand out for ingrained attitudes of sexism. The barriers and support women find in NZ are likely to be similar to those found in many countries.

**Figure 1.**
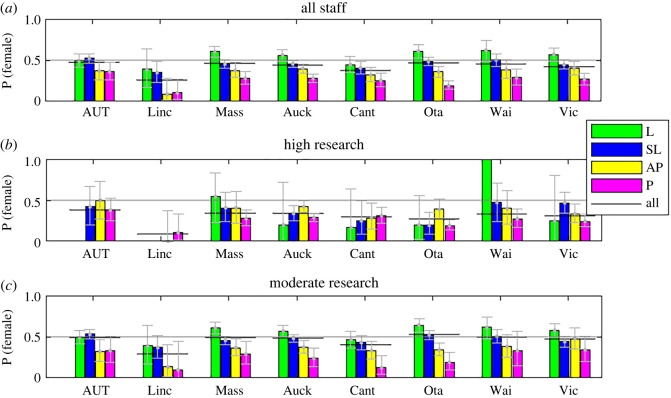
Proportion of women staff at each rank in NZ universities in 2018. Error bars show the binomial 95% confidence interval. Solid black lines show the proportion over all staff entered into the 2018 PBRF assessment. Grey horizontal line shows 50%. (AUT, Auckland University of Technology; Linc, Lincoln University; Mass, Massey University; Auck, University of Auckland; Cant, University of Canterbury; Ota, University of Otago; Wai, University of Waikato Vic, Victoria University of Wellington).

In 2018, there was no NZ university with equal representation of women across all academic levels though AUT was the closest at 47.5% women (Black lines figure 1*a*). Considering only individuals with high research activity (score≥ 500) (figure 1*b*, black lines), the proportion of women drops below 40% in all institutions. Considering only individuals with moderate research activity (score < 500) (figure 1*c*, black lines), there are proportionally more women but still only one institution has more than 50%, the University of Otago.

When we consider the proportion of women at each rank, a clear picture emerges. Including staff with any research activity at the lowest rank of L (figure 1*a*, green bars), we see that women are in the majority, i.e. over 50%, at five of the eight institutions. For seven of the eight institutions, the lowest rank of L has proportionally more women than any other rank. As rank increases the proportion of women drops steadily at every institution. At almost every institution, the probability of a P being a woman is around half the probability of a L being a woman.

If we only consider individuals with moderate research activity (figure 1*c*), the same picture of women's representation falling as rank increases is seen at every institution. However, considering only the higher performing individuals (figure 1*b*), the picture is less clear. Overall, the proportion of women is much lower but the difference in representation across ranks has no clear picture. This is possibly because of the smaller numbers of individuals involved, for example, at most institutions, there are fewer than 10 women with high activity at either of the lower ranks of L or SL. Electronic supplementary material, table S2 shows the number of men and women in each research activity group at each university.

Confidence intervals in figure 1 are the 95% confidence interval of the binominal parameter estimate.

## 5. Model

We use a simple Leslie Matrix population model with an annual time step to predict the number of individuals at each of the four ranks: L, SL, AP and P over time. The model inputs are the number of staff hired at each rank in an average year, the probability of staff at each rank being promoted to the next rank, and the probability of staff at each rank leaving. The key model assumption is that hiring, promotion and attrition rates in the future will match those seen in recent years. By iterating the model in time, or by calculating the steady state directly, we can predict the final equilibrium state of the system after inertial effects have played out if the current trends of hiring, promotion and attrition continue.

We parametrize and run all models separately for men and women, assuming that the populations are independent. For each scenario considered, we then combine the outputs for men and women and report the proportion of staff who are women at each rank either over time or when the system has reached equilibrium.

The model's three key inputs are calculated from data as follows:

### 5.1. Hiring

We calculate the expected number of individuals hired each year by rank, *H*. This is a simple mean across the data from 2005 to 2020. We assume the hiring rate is constant and not dependent on the size of the current population.

### 5.2. Attrition

We calculate the probability an individual will leave each year by rank. This is the mean of the proportion of people that left each year considering only individuals of that rank

$$L_{r}=\frac{1}{\mathrm{number}\;\mathrm{of}\;\mathrm{years}}\underset{\mathrm{years}}{\sum }\frac{\mathrm{number}\;\mathrm{of}\;\mathrm{individuals}\;\mathrm{at}\;\mathrm{rank}\;r\;\mathrm{who}\;\mathrm{left}\;\;\mathrm{in}\;\mathrm{yea}r\;y}{\mathrm{total}\;\mathrm{number}\;\mathrm{of}\;\mathrm{individuals}\;\mathrm{at}\;\mathrm{rank}\;r\;\mathrm{in}\;\;\mathrm{year}\;y}.$$

The total number of individuals at rank *r* in each year includes those who were hired in that year.

### 5.3. Promotion

We calculate the probability an individual will be promoted to the next rank in any particular year. This is the mean of the proportion of people promoted to a higher rank across all years

$$P_{r}=\frac{1}{\mathrm{number}\;\mathrm{of}\;\mathrm{years}}\underset{\mathrm{years}}{\sum }\frac{\mathrm{number}\;\;\mathrm{of}\;\mathrm{individuals}\;\mathrm{who}\;\mathrm{moved}\;\mathrm{from}\;\mathrm{rank}\;r\;\mathrm{to}\;\mathrm{rank}\;r+1\;\mathrm{in}\;\mathrm{year}\;y}{\mathrm{total}\;\mathrm{number}\;\mathrm{of}\;\mathrm{individuals}\;\mathrm{at}\;\mathrm{rank}\;r\;\mathrm{in}\;\;\mathrm{year}\;y}.$$

Finally, *S_r_* = 1 − *L_r_* − *P_r_* is the probability an individual will stay at that rank that year.

We assume that all three model inputs are constant over time. Data exploration showed that overall, this was true and the details of this are given in the Sensitivity analysis and model limitations section.

We combine these three parameters to get a matrix ***A*** which has diagonal elements *A_ii_* = *S_i_* and lower diagonal elements *A_i_*_+1,*i*_ = *P_i_*. The Leslie matrix model is given by

$$\underline{R}(Y+1)=\underline{H}+A\underline{R}(Y),$$

where *R_i_*(*Y*) is the number of individuals at rank *i* in year *Y*.

Demotions and double promotions, where an individual moves from rank *r* to rank *r* + 2, are non-existent in both the data and the model. The attrition rate from each rank is implicit in the ***A*** matrix. Attrition from rank *i* is $L_{r}=1-\sum_{j}A_{\;j,i}$, i.e. 1 minus the column sum of ***A***.

Promotion for an individual is independent of promotion for others both in the model and at UC which does not cap the number of individuals who can be promoted in one year. So we can consider any specific group separately of others.

We use the data to find estimates of ***A*** and *H* for the following groups:

— All individuals: women ***A****_W_*,H*_W_;* men ***A****_M_*, H*_M_*.
— Moderate research activity, i.e. with a maximum PBRF score between 2006 and 2018 of less than 500: women***A****_WL_*, H*_WL_*; men ***A****_ML_*, H*_ML_*.
— High research activity, i.e. with a maximum PBRF score between 2006 and 2018 of greater than or equal to 500: women ***A****_WH_*, H*_WH_*; men ***A****_MH_*, H*_MH_*.

We find the model equilibrium or fixed point, i.e. when R(*Y* + 1) = R(*Y*) to estimate the number of individuals at each rank in the future under current hiring, promotion and attrition rates. Provided all the diagonal elements of *A* are less than 1, i.e. there is a non-zero probability that an individual will leave every rank at some point either through promotion or attrition, the model will reach steady state, and the steady state is independent of the initial condition. If it exists, the steady state is stable, and it is the stationary distribution of the Leslie matrix [16]. For a particular group, for example high research activity staff, we run the model for men and women separately then combine the two outputs to determine the proportion of staff at each rank who are women.

The model assumes Hiring is constant each year. In reality, this will probably change, for example, when student numbers dropped significantly after a major earthquake in Christchurch in 2011, there was a brief hiring moratorium. However, the total number of new hires predominantly affects the total number of individuals employed and the time to reach steady state, the key model output of the proportion of staff who are women remains relatively unchanged.

## 6. Confidence intervals

We use bootstrapped confidence intervals to give an estimate of the robustness of the results. For each bootstrap simulation, we randomly choose 10% of the data population being considered and re-assigned their gender from man to woman or vice versa. We then recalculate all the parameter values using this modified data and rerun the model. Using 500 bootstrapped simulations, we then present the 95% range of the output.

## 7. Model fit

**Figure 2.**
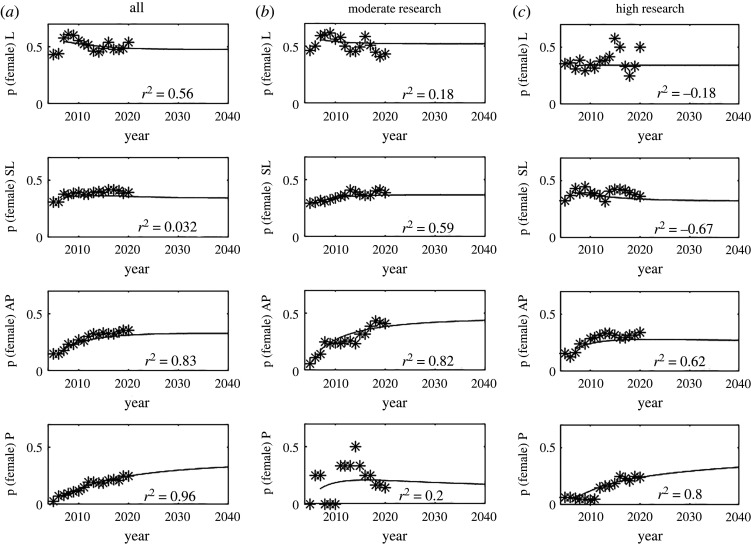
The model is often an excellent fit to the data with ***r***^2^ ≥ 0.8 in.many cases. Model output compared with data showing the proportion of women at each rank over time. The best fit is seen using the full dataset (column A). But using the smaller datasets of only staff with moderate (column B) or high (column C) research activity is still a good fit. The initial condition is the mean of the data between 2005 and 2010. The proportion of women staff at the Lecturer level (top row) has already reached steady state. At the Professorial level (bottom row) it will not be reached till shortly after 2040.

Figure 2 shows the model output. The coefficients of determination, *r*^2^, is calculated using

$$r^{2}=1-\frac{\sum {\left(y_{\mathrm{model}}-y_{\mathrm{data}}\right)}^{2}}{\sum {\left(y_{\mathrm{data}}-\bar{y}\right)}^{2}}.$$

A value of *r*^2^ ≈ 0 shows a correlation no better than the mean value, whereas *r* ≈ 1 is almost perfect correlation between model prediction and data. Negative values of *r*^2^ show a correlation worse than the mean value of the data. The data at the start of the time period, particularly for the L group is relatively noisy, to counteract this the initial condition for the model, i.e. the number of individuals in each group in 2005 is the mean of the data between 2005 and 2010. When we consider staff with all research activity scores (figure 2, column A), the lowest rank of L is dominated by new hires and the model makes a very poor prediction. The number of new hires varies considerably throughout the data period, particularly as it spans the time of a major earthquake in Christchurch which had a significant effect on university student numbers and hence hiring policies. Conversely, the higher ranks that are more likely to be joined through promotion and have fewer new hires, show a high correlation between model and data. Overall the model predicts that a steady state in the proportion of women staff has already been reached at the lowest rank of L at around 48% women. SLs are not far behind and are predicted to very soon reach a steady state at 34% women. APs are expected to reach steady state in the next 10 years at 33% women and in a little over 20 years around 36% of Ps are expected to be women.

This same pattern is seen when we parametrize the model with data from the moderate research activity group. The lowest rank shows poor correlation due to the domination of highly variable hiring and correlation improves as we move up the ranks of SL and AP. However, the correlation between data and model for the highest rank of P is very poor. This is due to the very small number of individuals in this group, rarely more than 10. The predicted time to reach parity for this demographic is similar to that for the population as a whole and the final steady states are L: 52%, SL: 36%, AP: 45%, P: 15%.

Finally, we consider those individuals with high research activity. Again, correlation is poor at the lower ranks and good at the highest ranks and steady state is reached in a similar time frame for the lower ranks. However, (not shown here) the proportion of Ps who are women is predicted to continue to rise and only reaches equilibrium after 2060. The prediction for the final proportion of women staff at each level is L: 34%, SL: 32%, AP: 27%, P: 43%.

**Figure 3.**
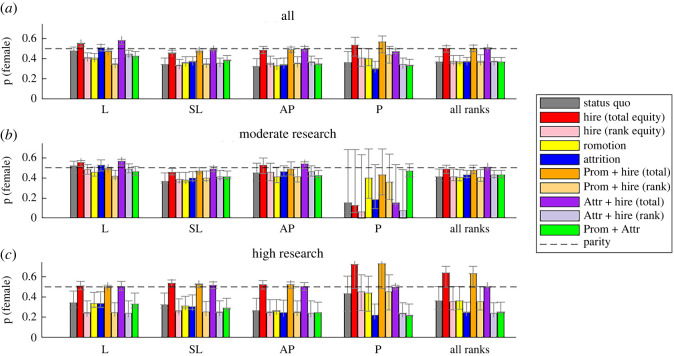
Hiring more women will have the biggest overall effect on improving gender representation but improving promotion equity will significantly improve the proportion of women at the highest rank. Proportion of women predicted to be at each rank after all inertial effects have played out under a range of equity scenarios. (*a*) All individuals. (*b*) Only individuals with moderate research activity. (*c*) Only individuals with high research activity. Error bars are 90% bootstrapped ranges.

Bootstrap confidence estimates for the final steady state are shown in figure 3.

## 8. Management scenarios

By separating the three key drivers of gender equity—hiring, attrition and promotion—the model provides a useful tool to explore which management strategies will have the most effect on improving the number of women at each Rank. We consider a range of scenarios where each of the key drivers is altered, either alone or in conjunction with another driver. This is done by modifying *H* and/or ***A*** to align with the management scenario being considered. The modified parameter is denoted by either $\hat{H}$ or $\hat{A}$. For each scenario, we calculate the long-term equilibrium solution and report the fraction of women at each rank and compare with the status quo solution presented in figure 2.

### 8.1. Hiring equity

Gender equity in hiring can be achieved in one of two ways, *Rank equity*, where the number of women hired is not changed but they are hired at a rank distribution comparable with men hired, or *Total equity* where the number and rank distribution of women hires matches that of men.

(1) Rank equity—this keeps the total number of women hired unchanged but distributes them so they match the rank distribution of the men hired, i.e. ${\underline{\hat{H}}}_{W*}=\alpha {\underline{H}}_{M*}$ where *α* is the total number of women hired each year divided by the total number of men hired to keep the total number of women hired unchanged.
(2) Total equity—this takes the total number of hires (men plus women) and assigns half to be men, and half to be women. This is done separately at each rank to keep the overall rank distribution unchanged, i.e. ${\underline{\hat{H}}}_{W*}={\underline{\hat{H}}}_{M*}=$ (H_M__*_ + H_W__*_)/2.

*Promotion equity only* women's rate of leaving remains unchanged but those that stay are as likely to be promoted as men. To model this, we keep the column sums of ***A***_W__*_ fixed, i.e. attrition is unchanged, but change the ratio of *A_ii_*:*A_i_*_,*i*+1_ to match the men's ratio for the same rank.

*Attrition equity only* women's rate of leaving is changed to match the men's rate. Here we change the column sum of ***A***_W__*_ to match the equivalent column sum of ***A***_M__*_ but keep the ratio of *A_ii_*:*A_i_*_,*i*+1_ fixed.

We examine the effect of each equity intervention both individually and in combination with one of the two other equity interventions. When Hiring equity is combined with either Promotion or Attrition equity this results in independent changes of both *H* and *A* as described above. When the promotion and attrition equity strategies are combined, i.e. women have the same promotion and attrition rates as men, the result of both changes on the *A* matrix results in $\;{\hat{A}}_{W*}=A_{M*}$.

In all scenarios bar Total hiring equity, the model for men staff remains unchanged. We consider each of these scenarios for the whole population, staff with moderate research activity and high research activity separately.

Figure 3 shows the output of long-term gender representation by Rank for each scenario. The rightmost set of bars also shows the gender split for al ranks combined. In figure 3*a*, we consider the entire population. There are three scenarios that bring the gender representation at each rank closest to parity; all three include the hiring ‘lever', called ‘Total Hiring Equity', i.e. half of new hires at every level are female.

Figure 3*b* shows that under the status quo women with moderate research activity continue to dominate in the lowest ranks and are grossly under-represented in the highest rank of P in comparison with moderately active men under most scenarios. The four scenarios that have the most effect on this specific area of under-representation all include promotion equity.

Finally, figure 3*c* shows women with high research activity will continue to be very under-represented at the three lower ranks under the status quo. All scenarios that remedy this involve Total hiring equity rather than promotion or attrition equity.

## 9. Sensitivity analysis and model limitations

In splitting the population by research activity level, we used the highest research performance score an individual had achieved. This method will give a good estimate of long-term research achievement for older academics who have probably reached peak performance score, but might underestimate that of some younger academics whose research has not yet reached its full potential. To correct for this, we tested a different cut-off for research activity based on age. Using an individual's oldest age while employed by UC, we kept the same cut off (500) for any academic over 45 and used a lower cut off (400) for younger academics. This reclassified an additional 35 individuals as high research activity (from 220 using the age-free classification), including 12 women. It made no noticeable change to the results.

Other definitions of high and moderate performance were also tested. For example, taking an individual's mean score rather than maximum score; using a different research score cut-off; different age cut-offs for younger academics. Provided the definition of high research activity was within a reasonable set of limits that included approximately 1/3 of the population the overall results were not affected.

The model assumes that the number of individuals hired each year does not depend on the current number of staff. This mirrors the data where annual hiring showed no dependence on staff numbers. However, we recognize that hiring is unlikely to be constant in reality. It often depends on student numbers which are affected by a wide range of factors from political whims to natural disasters. For these reasons, we made the first order assumption that hiring is constant but for these same reasons we did not consider total number of staff to be a reliable model output. This is highly dependent on actual hiring numbers whereas the reported output of fraction of staff who are women is more robust.

Our calculations of the model parameters assume that hiring, attrition and promotion rates have not changed for the duration of the dataset. To test this assumption, we used a regression model (linear for the number of individuals hired each year, i.e. $\mathrm{Number}\;\mathrm{of}\;\mathrm{new}\;\;\mathrm{Hires}\;\tilde{\;}\mathrm{Year}$, logistic for the probability an individual is either promoted or leaves each year, i.e. $\mathrm{logit}(P(\mathrm{individual}\;\mathrm{is}\;\mathrm{promoted}\;\mathrm{from}\;\mathrm{Rank}\;i)\tilde{\;}\mathrm{Year}$) to test the effect of year on each of the three parameters separately. We ran the models separately for each gender-rank data subset and for all ranks combined, then for the three research activity groupings (all, moderate, high), i.e. three parameters, five ranks (L, SL, AP, P, All), two genders, three research activity levels (all, moderate, high): 90 regressions. About 20% of models indicated a small change over time (see electronic supplementary material, table S2 for full results). Many of these are likely to be type I error but they do indicate a possible small increase in promotion rates since 2005, which is not considered in the model. As this increase affects men and women almost equally, it is unlikely to significantly change the results.

## 10. Discussion

We use Leslie matrices to compare the effectiveness of tweaking three drivers of gender equity in representation across university ranks—hiring, promotions and attrition—to ask which, and in what combination, might hold the most promise for creating a more equitable gender distribution across the rank structure from L to P. Through this comparison, we are also able to test, and reject, the demographic inertia hypothesis, that after a time lag we will see [9] and parity will come about without intervention. Instead of seeing the ranks shift towards equal gender representation by 2050 of their own accord as demographic inertia would predict, we see intervention is required. Comparing the three levers, we also find that which lever is the most effective depends on level of research activity.

For those with moderate research activity equity interventions must tweak the promotions lever in particular the highest rank of P. All of the four scenarios with the strongest observable effect to equalise the representation of men and women with moderate research scores at the P rank include tweaking the promotions lever. It is not surprising to see only a few moderately scoring men or women at P rank, because few academics with low research activity are hired at a Professorial level; and most moderately scoring Ps will have got there through internal promotions often for their Service or Teaching prowess. The surprising bit is that markedly more moderately scoring men make it to P than moderately scoring women. This is despite the international evidence that women become stronger in the teaching and administrative side of university employment [26], whether they want to or not [27]. Despite women's reported strength in the less research-intensive areas of university life, moderately scoring women do not seem to be promoted in the same way as men.

For the higher scoring group, equity interventions must pull the hiring lever. All of the scenarios that show equity improvement for the higher scoring group revolve around the simple solution of hiring more women. Improving their promotion success rate to match that of comparable men has almost no effect and changing their attrition rate actually makes representation worse, particularly at the highest rank of P. This suggests that women with high research activity are less likely to leave than men, a finding not seen in studies that do not split the population by research performance [28].

In our dataset, all of the scenarios that show equity improvement for the higher scoring group pull the simple lever of hiring more women. However, this hire more women strategy will only work if women are hired at the same rate as men **at each rank, i.e.** half of the new hires at L level are women, half of the new SLs are women, half of the new APs are women and half of the newly hired Ps are women.

On the topic of rank offered at the time of first employment, gendered bargaining behaviours and success rates might play a role. Some studies observe that women are less likely to ask for a higher rank or salary when hired, and when they do ask for more, women are less successful than men [29,30] others who say that when salary bargaining is explicitly allowed, women bargain as often as men [31]. Whatever the underlying cause, changing hiring patterns could make the sticky floor less sticky [32]. Our model shows that pulling this lever will have profound effects on equity in gender distribution across the ranks for all women and those with high research activity in particular.

The flip side to hiring is attrition. A common way for one institution to gain a high-ranking woman is for another institution to lose one. This path to P is via the metaphorical helicopter – changing jobs to move up the ranks. Our data from one university suggest that the helicopter doesn't carry many women to P and there would be over-representation of women at the professorial level if it did. We saw in figure 3*c* that changing women's attrition rates to match men's reduces the number of high research activity women Ps. This suggests that women's attrition is lower than men's in this group. Combined with the information that this group are relatively less likely to be hired than men (i.e. equitable hiring rates increases the number of women here) this further supports the idea that women cannot helicopter to P easily or often. International literature suggests that women are less likely to apply for jobs at other universities, perhaps due to stronger local ties and commitments [33]; but again it could be that women are applying, but not getting selected for the helicopter ride [29].

Our model highlights two key recommendations: (1) improve the promotion success rate for moderately research active women at the highest ranks and (2) hire more women with high research activity. The first recommendation is, in theory, simple and, with an institutional will, a way could be found. The second is more complex and raises the elephant in the room question of do these women exist? Why are women over-represented in the moderately scoring group and under-represented in the high scoring group. Without doing a national study of the research scoring system itself, that would almost necessarily involve violations of anonymity in a country of only 5 million, we cannot say with certainty whether or how the scoring system is biased or not. But the literature offers several explanations of why women might score lower at certain times of their lives, including child rearing responsibilities [34]. There is also the possibility that studies suggest women often do more teaching, administration and ‘non-promotable' tasks [27,35–37] which will depress research outputs and scores. And finally, there is the ‘double-whammy' theory of gender equity, in which universities ‘over-demand and under-reward women's teaching and service' meaning that women ‘suffer doubly in promotions from simultaneously researching less due to higher teaching and service expectations, while still failing to meet the burden of those higher expectations' [6].

## 11. Conclusion

We compared three levers that universities can pull to increase gender equity among staff across all ranks—hiring, promotions and attrition. We found changing hiring patterns increases equity patterns for high performing women, while changing promotions patterns does more for moderate performers. These findings have management implications for universities seeking to increase gender equity in representation across the ranks. HR departments should employ different interventions for different populations. But, since those populations might be indistinguishable at first hiring as it is hard to predict a prospective employee's score 20 years hence, the wise HR department would tweak both the hiring and the promotions levers. While a focus on reducing attrition among women should never be discouraged, perhaps a focus on empowering women who might have moderate research scores to get promoted would go further to achieving equity.

## Data Availability

For confidentiality reasons, the UC dataset is not publicly available. The authors were granted access privileges to the anonymized data, under strict non-disclosure agreements by the vice-chancellor of the university. The TEC data used in this study are owned by a third-party organization (Tertiary Education Commission, New Zealand). The authors were granted access privileges to the data, under strict non-disclosure agreements, by the TEC under New Zealand's Official Information Act 1992. The Official Information Act 1992 facilitates New Zealanders' access to government records, through a formal information request. All NZ citizens and residents may make such requests, under the following guidelines: https://www.dia.govt.nz/Official-Information-Act-requests. Both datasets pertain to thousands of people's employment; hence are strictly private and highly sensitive. Due to ethical and privacy restrictions, a de-identified dataset cannot be made publicly available. However, interested researchers will be able to replicate the authors' methods by using the information provided in methods, and applying it to any similar data. Interested researchers are invited to contact the corresponding authors to discuss access to data. Supplementary material is available online [38].
